# Diversity and Current Classification of dsRNA Bacteriophages

**DOI:** 10.3390/v15112154

**Published:** 2023-10-25

**Authors:** Sari Mäntynen, Meri M. Salomaa, Minna M. Poranen

**Affiliations:** Molecular and Integrative Biosciences Research Programme, Faculty of Biological and Environmental Sciences, University of Helsinki, 00014 Helsinki, Finland; meri.salomaa@helsinki.fi (M.M.S.); minna.poranen@helsinki.fi (M.M.P.)

**Keywords:** bacteriophage, dsRNA virus, cystovirus, virus taxonomy

## Abstract

Half a century has passed since the discovery of Pseudomonas phage phi6, the first enveloped dsRNA bacteriophage to be isolated. It remained the sole known dsRNA phage for a quarter of a century and the only recognised member of the *Cystoviridae* family until the year 2018. After the initial discovery of phi6, additional dsRNA phages have been isolated from globally distant locations and identified in metatranscriptomic datasets, suggesting that this virus type is more ubiquitous in nature than previously acknowledged. Most identified dsRNA phages infect *Pseudomonas* strains and utilise either pilus or lipopolysaccharide components of the host as the primary receptor. In addition to the receptor-mediated strictly lytic lifestyle, an alternative persistent infection strategy has been described for some dsRNA phages. To date, complete genome sequences of fourteen dsRNA phage isolates are available. Despite the high sequence diversity, similar sets of genes can typically be found in the genomes of dsRNA phages, suggesting shared evolutionary trajectories. This review provides a brief overview of the recognised members of the *Cystoviridae* virus family and related dsRNA phage isolates, outlines the current classification of dsRNA phages, and discusses their relationships with eukaryotic RNA viruses.

## 1. Introduction

Bacteriophages are highly abundant in the biosphere and play a fundamental role in modulating the ecology and evolution of global bacterial communities. While the diversity and role of bacteriophages with DNA genomes have been widely described in ecosystems, the biological significance of RNA phages has often been overlooked. Double-stranded (ds)DNA bacteriophages are classified by the International Committee on Taxonomy of Viruses (ICTV) into almost 50 virus families, while only one family of dsRNA bacteriophages is recognised: the family *Cystoviridae,* currently comprising seven species (Table 1). In contrast to dsDNA phages that have been isolated from a wide range of bacterial hosts, the host range of classified dsRNA phage isolates is relatively narrow. However, the recent isolation of additional dsRNA phages and metatranscriptome analyses of viral sequences from different environments show that dsRNA phages are more widespread in nature than previously anticipated [1,2,3,4,5,6,7,8]. This review outlines the diversity and current classification of dsRNA phages. We acknowledge the extensive literature on Pseudomonas phage phi6; however, the emphasis here is on the dsRNA phage diversity and therefore details of the structure and life cycle of phi6 are only included where comparable information is available for other dsRNA phages.

## 2. Discovery of dsRNA Phages

**Figure 1:**
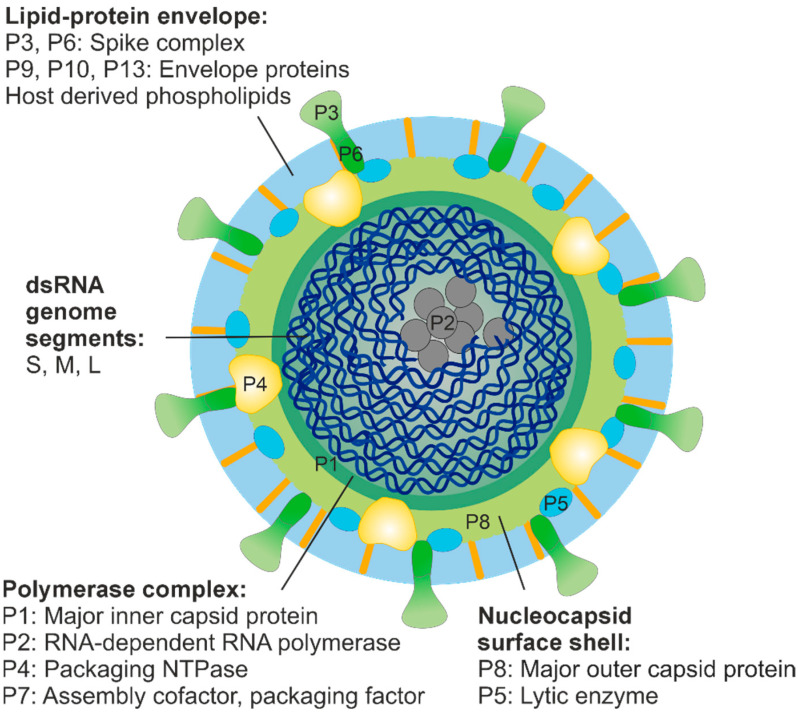
Schematic presentation of phi6 virion. The size of the enveloped virion is about 85 nm [27]. The lipid-protein envelope encloses the nucleocapsid, which comprises two concentric protein shells: the nucleocapsid surface shell and the polymerase complex. Hexamers of the packaging NTPase P4 are attached on the icosahedral five-fold vertices of the polymerase complex and protrude through the nucleocapsid surface shell [28]. The spooled dsRNA genome is tightly packed inside the polymerase complex [29] together with about ten copies of the viral polymerase subunit P2 [30].

Half a century has passed since Pseudomonas phage phi6, the example virus of the *Cystoviridae* virus family, was isolated from *Pseudomonas*-infested bean straw in Nebraska, USA [9]. Phi6 differed from all other known bacteriophages in two fundamental aspects: it was the first described phage that had a lipid envelope surrounding a polyhedral capsid and a segmented dsRNA genome (Figure 1). Since its discovery, phi6 has been a popular model virus, contributing significantly to our understanding of the structure, assembly and genome replication mechanisms of dsRNA viruses [18]. This research has also resulted in biotechnological applications, and phi6 is currently being used as a tool to produce high-quality dsRNA molecules to combat viral diseases through RNA interference [19,20,21,22]. Moreover, cystoviruses have been recognised as surrogate models for pathogenic human enveloped viruses such as coronaviruses, influenza and Ebola viruses [23,24,25,26].

Phage phi6 remained the only known dsRNA phage for more than 25 years, until eight additional dsRNA phages (Pseudomonas phages phi7–phi14) were isolated from leaves of various plants in the USA [10]. Three of these phage isolates (Pseudomonas phages phi8, phi12 and phi13; species *Cystovirus phi8*, *Cystovirus phi12* and *Cystovirus phi13*, respectively; Table 1) have been subjected to a more detailed characterisation [10,11,12,13,14] (Table 2). A decade later, Pseudomonas phage phi2954 (species *Cystovirus phi2954*), showing notable similarities to phage phi12, was isolated and characterised by the same research group [15]. Additional dsRNA phage isolates were obtained by sampling clover leaves and green beans at different locations in the USA [31,32], but these virus isolates have not been characterised beyond partial sequencing. Over the past decade, the sampling of assorted environmental sources in Europe and Asia yielded additional dsRNA phages: Pseudomonas phage phiNN (species *Cystovirus phiNN*) was isolated from a fresh water sample in Finland [16], whereas the isolation source of Pseudomonas phage phiYY (species *Cystovirus phiYY*) was hospital sewage in China [17].

In recent years, more novel dsRNA phage isolates have been reported, including phage phiNY [1] and phage phiZ98 [8] that were isolated in China from fermented sour soup and horse manure, respectively, as well as five related phage isolates, CAP3, CAP4, CAP5, CAP6 and CAP7 (hereafter collectively phages CAP3–7), isolated from duck and turkey faeces in the USA [3]. Moreover, metatranscriptome surveys have uncovered a plethora of cystovirus-like sequences from various environments [2,4,5,6,7]. For instance, cystovirus-like partial viral genomes were detected in metatranscriptomic datasets derived from the Zodletone sulphur spring [4], animal faeces, and soil and lake/pond sediments across China [2], associated with poultry red mite *Dermanyssus gallinae* transcripts [33] and global ocean RNA sequence datasets provided by the Tara Oceans expeditions [7]. These new discoveries demonstrate that dsRNA phages have adapted to varying habitats in globally distant locations and are more widespread and abundant than previously recognised.

**Table 2:** Key methods utilised for the initial characterisation of dsRNA phage isolates.

	Method ^1^	Phi6	PhiNN	Phi2954	Phi8	Phi12	Phi13	PhiYY	PhiNY	PhiZ98	CAP3 ^2^
Genome type	Gel electrophoresis	[34]	[16]	[15]	[11]	[15]	[14]	[17]	[1]	[8]	[3]
	RNase sensitivity	[34]						[17]	[1]	[8]	[3]
Virion morphology	TEM imaging	[9,35]	[16]					[17]	[1]	[8]	[3]
	Cryo-EM/Cryo-ET imaging	[27,28]		[36]	[28]	[36,37]					
Virion composition	PAGE of structural proteins	[38]	[16]	[15]	[11]	[37]	[14]	[17]		[8]	[3]
	Chloroform sensitivity	[9]	[16]	[15]	[10]	[10]	[10]	[17]	[1]	[8]	[3]
	Detergent sensitivity	[35]	[16]	[15]	[11]	[12,13]					[3]

^1^ TEM, transmission electron microscopy; cryo-EM, cryo-electron microscopy; cryo-ET, cryo-electron tomography; PAGE, polyacrylamide gel electrophoresis. ^2^ The representative member of CAP phages.

## 3. Diversity of dsRNA Phages

### 3.1. Host Range of dsRNA Phages

Pseudomonas phage phi6 was initially isolated using *Pseudomonas syringae* pv. *phaseolicola* HB10Y as the host strain, and since then several additional dsRNA phages have been isolated using hosts belonging to the genus *Pseudomonas*, mainly plant-pathogenic *P. syringae* (Table 1). Phage phiYY was the first dsRNA phage shown to infect the opportunistic human pathogen *Pseudomonas aeruginosa* [17], while phage phiZ98 can productively replicate in certain *P. aeruginosa* and *P. fluorescens* strains [8]. Phage phiNY was isolated from *Microvirgula aerodenitrificans,* an aerobic and heterotrophic denitrifier [1], and phages CAP3-7 were shown to infect *Acinetobacter radioresistens* bacteria [3]. Interestingly, a cystovirus-like partial viral genome has also been detected in metatranscriptomic datasets derived from a pure culture of Gram-positive *Streptomyces avermitilis* [4]. If experimentally confirmed, this would be the first known dsRNA phage infecting Gram-positive bacteria. Overall, the emerging data indicate that dsRNA phages have adapted to infect a variety of different host bacteria and may thus have a significant effect on different bacterial communities.

### 3.2. Host Cell Receptors for dsRNA Phages

Initial electron microscopic studies of phi6 revealed virions attached to the type IV pilus of the *P. syringae* host [9,35,39]. Further evidence on the dependence of phi6 infection on a functional, retractable pilus was obtained through analyses of non-piliated derivatives of the host that showed resistance to phi6 infection [40]. In addition to phi6, two other cystoviruses, phiNN and phi2954, have been shown to attach to or be dependent on the pilus of the *Pseudomonas* host [15,16]. Furthermore, the pilin protein, PilA, is required for the infection of *Acinetobacter* by the dsRNA phage CAP3 [3]. Instead, cystoviruses phi8, phi12 and phi13 were shown to infect non-piliated *Pseudomonas*, and more precisely strains which have a rough lipopolysaccharide (LPS) layer, while smooth LPS strains were resistant to these phages [10]. Moreover, core polysaccharides of LPS were recently identified as a receptor for cystovirus phiYY infection [41], and a defective LPS layer made *P. aeruginosa* and *P. fluorescens* strains susceptible to phiZ98 infection [8]. Thus, based on the current information, dsRNA phages have evolved to utilise either LPS or pilus components as their primary receptors on the host surface.

### 3.3. Infection Strategies of dsRNA Phages

Cystovirus isolates are generally regarded as virulent viruses that cause lysis of their host bacterial cells at the end of the reproduction cycle. However, some dsRNA phages can also establish an alternative infection cycle in which viral particles form and remain inside the host cell without integrating into the host chromosome or causing lysis. We have recently suggested this single-cell phenomenon be called a non-productive chronic infection, or a carrier cell, to distinguish it from the carrier state life cycle that should be reserved to describe population-level interactions between phages and their hosts [42]. Cystovirus carrier cells have been identified by detecting intracellular viral genomic dsRNA molecules or intracellular viral particles in constantly growing cell cultures [1,43]. This infection mode was initially reported for phi6 and its *P. syringae* host [43,44], but has also been observed to extend the host range of the phi13 derivative (carrying a kanamycin resistance gene) to *Salmonella typhimurium* and *Escherichia coli* strains [10]. A similar infection mode was also recently reported for dsRNA phage phiNY [1]. The persistent phiNY infection appeared to boost the growth of the *Microvirgula aerodenitrificans* host, suggesting a mutualistic, parasitic lifestyle for the phage [1]. Interestingly, the persistent infection mode of dsRNA phages resembles that of fungal dsRNA viruses, which do not produce extracellular infectious virions and are predominantly cryptic.

### 3.4. Organisation of the dsRNA Phage Virions and Structural Relationships with Eukaryotic RNA Viruses

The structures of different dsRNA phages, their component proteins and assembly intermediates have been analysed to varying degrees, phi6 and phi12 being currently structurally best characterised. However, the recognition of similar sets of genes in known dsRNA phage isolates (see Section 3.5) suggests that these viruses share a similar overall virion organisation. Nevertheless, emerging data also demonstrate some structural variation, especially on components related to host recognition and entry.

Images of dsRNA phages phi6, phi8, phi12, phi2954, phiNN, phiYY, phiNY, phiZ98, and CAP3 obtained using negative-stain transmission electron microscopy (TEM) [1,3,8,9,16,17,35], cryo-electron microscopy (cryo-EM) [27,28] or cryo-electron tomography (cryo-ET) [36,37] have depicted enveloped spherical virions. Further evidence for the presence of lipid components in the virion of different dsRNA phages has been obtained via detergent or organic solvent sensitivity analyses (Table 2). To date, these are the only known RNA bacteriophages with a membrane envelope.

Subsequent characterisation of phi6 and phi8 virions using cryo-EM revealed spikes of 2 nm and 7 nm, respectively, on the envelope surface [28]. Based on earlier analyses of P3-deficient phi6 virions, these structures represent host attachment spikes formed by multimeric complexes of P3 [27]. Later, cryo-ET and three-dimensional reconstruction were used to identify toroidal or elongated structures on the surface of phi12 and phi2954 virions, respectively [36,37]. A recombinant phi12 phage, comprising the S and L segments from phi12 and the M segment from phi2954, was produced by reverse genetics. The recombinant phage displayed phi2954-type envelope surface structures and also had the host specificity of phi2954 [36,37].

The viral envelope of dsRNA phages surrounds an icosahedrally symmetric nucleocapsid (Figure 1). Two structural layers were identified in these particles in the early EM studies on phi6 virions [45,46], namely the nucleocapsid surface shell and the polymerase complex (or virion core). Based on high-resolution cryo-EM imaging and three-dimensional reconstruction analyses on phages phi6 and phi12, the nucleocapsid shell, made of protein P8 trimers, is arranged into an incomplete icosahedral *T* = 13 lattice interrupted at the icosahedral five-fold symmetry positions by P4 complexes protruding from the underlying polymerase complex layer [47,48,49,50]. However, cystovirus phi8 deviates from this basic structure as it is lacking a distinct nucleocapsid shell [28]. Phi6 utilises the P8 layer for the binding and penetration of the host plasma membrane during entry [51,52,53]. In phi8, these essential functions are likely mediated by the components of the polymerase complex as purified phi8 core particles can be used to infect host cell spheroplasts [54]. The presence or absence of a distinct nucleocapsid surface shell for plasma membrane penetration in other related dsRNA phages is currently an open question, which requires further investigation.

The polymerase complex of phi6 is composed of proteins P1, P2, P4 and P7 (Figure 1), and it mediates the replication and transcription of the viral dsRNA. Dimers of the major inner capsid protein (MCP) P1, arranged on an icosahedral *T* = 1 lattice, have been depicted in the empty and/or genome-containing polymerase complex particles of phi6, phi8 and phi12 using cryo-EM techniques [28,48,49,50,55,56,57]. Interestingly, similar capsid organisation (referred as “*T* = 2”) has been described for a variety of fungal and plant dsRNA viruses (e.g., members of the families *Partitiviridae, Totiviridae, Chrysoviridae, Quadriviridae* and *Megabirnaviridae*), picobirnaviruses, as well as for the inner capsids of animal and plant dsRNA viruses of the *Reovirales* order [58,59], but not for any other viruses. This observation has promoted a hypothesis of a shared common ancestor for the dsRNA virus capsids [18,60].

The structure of the phi6 polymerase subunit P2 was described by Butcher et al. in 2001. This study presented the first high-resolution structure for an RNA-dependent RNA polymerase (RdRp) from a dsRNA virus [61]. The phi6 P2 structure displayed striking structural similarity with the hepatitis C virus RdRp, suggesting a shared evolutionary origin for the polymerase subunit of a dsRNA phage and a eukaryotic positive-sense single-stranded (ss)RNA virus. During the past two decades, a number of high-resolution structures for additional viral RdRps have been deposited in the protein data bank, and structure-based computational comparison of these RdRps has provided evidence that the phi6 RdRp, as well as phi12 RdRp [62], share a high level of structural similarity with all currently structurally characterised viral RdRps [63].

Protein P4 is one of the best structurally and functionally characterised proteins across different cystoviruses. P4 is a molecular motor which, using energy from NTP hydrolysis, drives the packaging of the viral single-stranded genomic precursor molecules into preformed empty polymerase complexes [64]. High-resolution structures are available for P4 proteins of phi6, phi8, phi12, phi13 and phiYY [65,66,67]. Despite some structural variation, all these proteins form ring-like hexameric complexes that structurally resemble RecA-type ATPases [65].

### 3.5. Genomes of dsRNA Phages

Each recognised member of the *Cystoviridae* family and the related dsRNA phage isolates (Table 1) has a genome that is divided into three dsRNA segments, designated according to their size as L (large, 6.3–7.1 kb), M (medium, 3.6–4.7 kb) and S (small, 2.3–3.2 kb) (Figure 1). The total genome size ranges between 12.7 (phi2954) and 15.0 kb (phi8). Based on comparative genomic analyses, some dsRNA phage isolates are close relatives of Pseudomonas phage phi6, whereas others are more distantly related [68]. For instance, Pseudomonas phages phiNN and phi6 share a relatively high-nucleotide sequence identity (80%, 55% and 84% nucleotide sequence identity between their L, M and S segments, respectively; [16]), whereas no apparent nucleotide sequence similarity has been reported between phiNY and other cystoviruses (or any other phage) [1].

Genome organisations of currently classified cystoviruses (Table 1) as well as the recent six dsRNA phage isolates, phiZ98 and CAP3–7, are similar. In each genome segment, genes are clustered into functional groups: the L segment contains proteins forming the polymerase complex (MCP P1, RdRp P2, packaging NTPase P4 and assembly factor P7), the M segment encodes proteins required for host recognition and host outer membrane penetration (P3 and P6), and the S segment encodes the nucleocapsid shell protein (P8), the major membrane protein (P9), putative membrane morphogenetic factor (non-structural protein P12) and the lytic protein (P5) [3,8,68]. Instead, most of the predicted open reading frames of phiNY encode proteins of unknown function, and only the RdRp and MCP genes in the L segment, as well as the glycoside hydrolase gene in the S segment, could be predicted [1], meriting further study. Nevertheless, in all cases the coding regions in the genome segments of dsRNA phages are flanked by non-coding regions. Based on phi6 studies, these non-coding regions are required for genome packaging and replication [69,70,71,72]. Despite the high level of gene synteny among the currently classified cystoviruses, additional open reading frames with unknown functions have been identified in some cases [68]. Furthermore, a specific function may be attributed to one or several cystoviral protein products, as seen in the P3 host recognition spike complex: cystoviruses phi6, phi2954 and phiNN possess a spike complex that is composed of a single P3 protein or its multimer [15,16,73], whereas the spike complex of cystoviruses phi8, phi12, phi13 and phiYY is heteromeric, consisting of two or three distinct viral proteins [11,13,14,17]. Moreover, recent metatranscriptomic surveys indicate that dsRNA phages may have acquired different types of lytic enzymes during their evolution. While the recognised members of the *Cystoviridae* encode lytic transglycosylases belonging to the lysozyme superfamily, putative N-acetylmuramoyl-L-alanine amidase, metallopeptidase (families M15 and M23), lipase and L-alanyl-D-glutamate endopeptidase genes were identified in some cystovirus-like contigs [5]. The diversity of lysis genes in these putative dsRNA phages could reflect an expanded host range.

## 4. Current Taxonomic Classification of dsRNA Phages

The phi6 group and phage phi6 were initially recognised by the ICTV in 1976 [74] and the *Cystoviridae* family was introduced in 1978 [75]. To date, the *Cystoviridae* virus family includes a single genus, *Cystovirus* (previously the phi6 group), which has seven virus species: *Cystovirus phi6* (type species), *Cystovirus phi8*, *Cystovirus phi12*, *Cystovirus phi13*, *Cystovirus phiNN*, *Cystovirus phi2954* and *Cystovirus phiYY* ([68,76]; Table 1). These virus species were grouped together due to the notable similarities the representative isolates share in the overall virion structures (see Section 3.4) and genome (see Section 3.5), even though the degree of nucleotide sequence identity between the genomes of these phages is relatively low (<50%, except for phi6 and phiNN, which have a higher nucleotide sequence identity; [16,68]). A 95% nucleotide sequence identity is currently the criterion for demarcation of species in the *Cystoviridae* family. Six related, unclassified viruses (phi7, phi9–phi11, phi14 and phiNY; Table 1) are currently also listed under the *Cystoviridae*, but are not yet officially classified. Furthermore, phiZ98 and phages CAP3-7 have been suggested to be included into the *Cystoviridae* family based on genetic and structural similarities [3,8] (Table 1).

**Figure 2:**
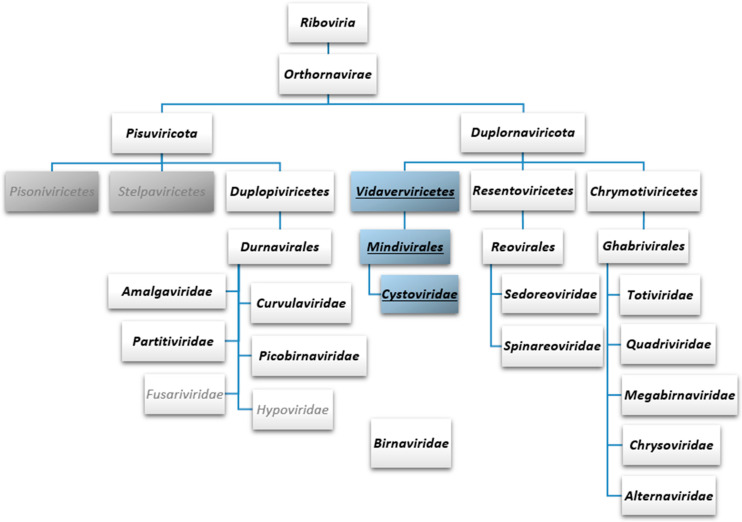
Taxonomy of eukaryotic and bacterial dsRNA viruses currently recognised by the International Committee on Taxonomy of Viruses. Two of the five phyla under the kingdom *Orthornavirae* are depicted. These two phyla, *Pisuviricota* and *Duplornaviricota*, comprise all the current dsRNA virus families, except *Birnaviridae,* which has not been assigned to any phylum. All the three classes of *Duplornaviricota* (*Vidaverviricetes*, *Resentoviricetes* and *Chrymotiviricetes*) comprise solely dsRNA viruses, while *Pisuviricota* contains both ssRNA (two classes; grey boxes) and dsRNA viruses (class *Duplopiviricetes*). dsRNA virus taxa are in bold, dsRNA phage taxa are underlined and in blue boxes. ssRNA virus families under *Duplopiviricetes* are in grey font.

ICTV has recently introduced additional higher-order ranks for the taxonomic classification of viruses and is moving towards classification which reflects the true phylogenetic relationships instead of grouping viruses based on the host specificity, morphological features or, e.g., disease symptoms [77,78]. The polymerase gene (either encoding RdRp or reverse transcriptase) has been selected as a hallmark gene to be used in the phylogenetic analyses and classification of RNA viruses. RNA viruses encoding an RdRp have been assigned into the kingdom *Orthornavirae* within the realm *Riboviria* (Figure 2). The kingdom *Orthornavirae* is subdivided into five officially recognised phyla: *Duplornaviricota, Kitrinoviricota, Lenarviricota, Negarnaviricota and Pisuviricota* [77]. In the current taxonomy, dsRNA viruses are assigned into phylum *Duplornaviricota* or *Pisuviricota* (Figure 2). The *Cystoviridae* virus family (class: *Vidaverviricetes,* order: *Mindivirales*) belongs to the phylum *Duplornaviricota*, together with the members of classes *Resentoviricetes* and *Chrymotiviricetes* comprising the majority of the known animal dsRNA viruses (e.g., rotavirus and bluetongue virus) as well as several important fungal and plant dsRNA viruses (e.g., Saccharomyces cerevisiae virus L-A and rice dwarf virus, respectively). Other dsRNA viruses, such as the amalga-, curvula, partiti- and picobirnaviruses are classified into the phylum *Pisuviricota* (Figure 2). This initial taxonomic classification of RNA viruses is based on a single holistic sequence-based comparison of viral RdRp sequences [79]. However, recent sequence-based RdRp phylogenetic analysis shows grouping of cystoviral RdRp sequences with RdRps of dsRNA viruses within phylum *Pisuviricota* [5]. Different higher-order grouping has also been obtained via structure-based phylogenetic analysis of viral RdRps [63].

## 5. Concluding Remarks and Future Research Directions

The year 2023 marks the 50th anniversary of the discovery of phage phi6, the first described enveloped dsRNA bacteriophage [9]. Phi6 maintained its unique status among bacteriophages for more than 25 years, until the isolation of eight additional dsRNA phages [10]. Since then, more related dsRNA phages have been isolated from environmental samples in the USA, China and Finland [1,3,8,15,16,17,31,32] and identified in metatranscriptome surveys [2,4,5,6,7]. The complete nucleotide sequences of 14 dsRNA phage isolates are currently available (Table 1). Based on current knowledge, these virus isolates share key physical, structural and genome characteristics. To date, only seven of these dsRNA phage isolates have been officially classified by the ICTV, suggesting an urgent need to update and refine the taxonomy of dsRNA phages.

In addition to the 14 fully sequenced dsRNA phages, numerous additional dsRNA phage isolates have been partially sequenced [10,31,32]. Whole genome sequencing of these isolates is needed to obtain a more comprehensive view of the genetic diversity among dsRNA phages. Such information would also support further classification of dsRNA phages under the order *Mindivirales* and family *Cystoviridae.* The higher order classification of RNA viruses will likely also be revised in the future. Here, the metagenomic surveys can provide important information on the sequence diversity of viral RdRp genes, supporting the classification of viruses, especially in the lower taxonomic ranks (up to order or even class). However, implementation of structure-based approaches is likely needed to enable prediction of deep evolutionary relationships of fast-evolving viral genes. Such information is required to support the assignment of the higher-order taxonomic ranks (e.g., phyla).

Culture-independent high-throughput RNA sequencing studies (aimed to identify new RNA viruses or reveal the transcriptomes of cellular organisms) have the potential to transform our understanding of the diversity, abundance and taxonomy of (ds)RNA viruses. However, genetic diversity of dsRNA phages (as exemplified by phage phiNY having no nucleotide sequence similarity with other phages) complicates their detection by metatranscriptomic analyses [1]. Thus, we need to acknowledge the importance of the isolation and characterisation of new (ds)RNA phages from different environments, including medical samples.

The limited host range of currently classified dsRNA phage isolates likely reflects the biased isolation method in which previously identified cystovirus host strains are used in phage enrichment. Furthermore, most dsRNA phages have been isolated using plaque assay. PhiNY is an exception as its genome was initially identified utilising CF11 cellulose, which is a method used to isolate fungal RNA viruses and does not require plaque production [1]. The use of this approach enabled detection of a non-lytic phage, highlighting the importance of harnessing alternative virus identification methods.

The alternative non-lytic life cycles of dsRNA phages (i.e., the carrier cell infection) require further studies. More information is needed on the molecular mechanisms, including essential phage and host factors, as well as environmental cues supporting the formation of carrier cell interaction between a dsRNA phage and its host. Phi6 is likely the best model to elucidate the genetic background of this phenomenon. The possibility to establish phi6’s carrier state using reverse genetic methods supports these analyses and has also paved the way for development of bacterial dsRNA production systems for heterologous sequences, facilitating development of novel RNA-based crop protection strategies where high quantities of high-quality dsRNA are needed [19].

The lytic power of dsRNA phages could potentially be harnessed to combat bacterial infections. The natural hosts of phi6 (and other dsRNA phages), *P. syringae* pathovars, infect a wide range of important crop plants globally, causing severe economic losses worldwide. Conventional treatments to control *P. syringae* infections are often inefficient or toxic to the environment, necessitating the search for new approaches. The potential of phi6 in biological control of plant bacterial diseases has been recently evaluated through in vitro studies [80], where the stability of the phage was analysed under variable environmental conditions. Furthermore, the antimicrobial potential of phiYY against *P. aeruginosa* infections has been under investigation [17,41], and the first in-human application of phiYY in the treatment of chronic lung infection by antibiotic-resistant *P. aeruginosa* has been reported [81]. However, application of dsRNA phages as biocontrol agents in agriculture or for therapy of human diseases warrants better understanding of the host range, host shift potential and the alternative non-lytic infection modes of these phages.

## Figures and Tables

**Table 1:** dsRNA phage isolates with complete genome sequence information described to date.

Virus Species ^1^, Phage Isolate	GenBank Accession Number (Segments L, M and S)	Isolation Host	Isolation Source, Country	Reference
*Cystovirus phi6*, Pseudomonas phage phi6	M17461, M17462, M12921	* Pseudomonas syringae * pv. *phaseolicola* HB10Y	* Phaseolus vulgaris * (common bean), USA	[9]
*Cystovirus phi8*, Pseudomonas phage phi8	AF226851, AF226852, AF226853	* P. syringae * pv. *phaseolicola* LM2333	* Pisum sativum * (pea), USA	[10,11]
*Cystovirus phi12*, Pseudomonas phage phi12	AF408636, AY039807, AY034425	* P. syringae * pv. *phaseolicola* LM2333	* Ocimum basilicum * (bacil), USA	[10,12,13]
*Cystovirus phi13*, Pseudomonas phage phi13	AF261668, AF261667, AF261666	* P. syringae * pv. *phaseolicola* LM2333	* Raphanus sativum * (radish), USA	[10,14]
*Cystovirus phi2954*, Pseudomonas phage phi2954	FJ608823, FJ608824, FJ608825	* P. syringae * pv. *phaseolicola* LM2489	* Raphanus sativum *, USA	[15]
*Cystovirus phiNN*, Pseudomonas phage phiNN	KJ957164, KJ957165, KJ957166	* Pseudomonas * sp. B314	Lake water, Finland	[16]
*Cystovirus phiYY*, Pseudomonas phage phiYY	KX074201, KX074202, KX074203	* Pseudomonas aeruginosa * PAO38	Hospital sewage, China	[17]
ND, Microvirgula aerodenitrificans phage phiNY	MW471133, MW471134, MW471135	* Microvirgula aerodenitrificans * LH11-4	Fermented sour soup, China	[1]
ND, Pseudomonas phage phiZ98	ON960064.1, ON960065.1, ON960066.1	* P. aeruginosa * SK98	Horse manure, China	[8]
ND, Pseudomonas phage CAP3	MZ558504, MZ558505, MZ558506	* Acinetobacter radioresistens * LH6	Duck faeces, USA	[3]
ND, Pseudomonas phage CAP4	MZ558507, MZ558508, MZ558509	* A. radioresistens * LH6	Turkey faeces, USA	[3]
ND, Pseudomonas phage CAP5	MZ558510, MZ558511, MZ558512	* A. radioresistens * LH6	Turkey faeces, USA	[3]
ND, Pseudomonas phage CAP6	MZ558513, MZ558514, MZ558515	* A. radioresistens * LH6	Turkey faeces, USA	[3]
ND, Pseudomonas phage CAP7	MZ558516, MZ558517, MZ558518	* A. radioresistens * LH6	Turkey faeces, USA	[3]

^1^ Virus species according to the International Committee on Taxonomy of Viruses; ND, not determined.

## Data Availability

No new data were created in the writing of the paper.
